# Pharmaceutical Industry in Vietnam: Sluggish Sector in a Growing Market

**DOI:** 10.3390/ijerph14090976

**Published:** 2017-08-29

**Authors:** Antonio Angelino, Do Ta Khanh, Nguyen An Ha, Tuan Pham

**Affiliations:** 1 Department of Economics and Management, University of Ferrara, Via Voltapaletto 11, 44121 Ferrara FE, Italy; 2 Vietnam Academy of Social Sciences, 1 Liễu Giai, Ba Đình, Hà Nội 119-405, Vietnam; dotakhanh@yahoo.com (D.T.K.); anhad4@yahoo.com (N.A.H.); famtuan@gmail.com (T.P.)

**Keywords:** pharmaceutical industry, Vietnam, emerging markets

## Abstract

Vietnam is a fast growing economy in the Asian region with a significantly high population (over 92 million in 2015). Although still expanding (about 1.1% on average during 2000–2015), the Vietnamese population is considered to be entering the ageing stage at a very high rate. The rapid expansion of the middle-income urban class and the ageing people ratio have dramatically pushed up the demand for healthcare goods, particularly in terms of pharmaceutical products. Since the early 1990s the government has addressed the necessities of rising demand for healthcare products by formulating a series of policies aimed at promoting the development of the pharmaceutical industry. However, the implementation of such policies does not seem to have been completely efficient given that the country still needs to import up to 90% of its pharmaceutical consumption. This paper aims to explore the development of the pharmaceutical industry during the years 1990–2015 and to identify a series of weaknesses in the government promotion of the industry. Future developments will also be discussed on how the Vietnamese pharmaceutical industry could increase its participation in the regional supply chain, which is currently being dominated by big players like India and China.

## 1. Introduction

Vietnam is among the highest growing markets in Asia for pharmaceutical products during 2011–2015 [1] and the country is expected to keep the pace for the next 20 years [2]. The overall market value of the industry in 2015 amounted to about 4.2 billion USD, and it is expected to reach 10 billion USD in 2020 [3]. According to BMI (Business Monitor International), Vietnam ranked 13/175 countries for the fastest growing global markets in drug spending in 2013 [4].

The demand for pharmaceutical products rapidly expanded as a consequence of high economic growth, rising income per capita, higher urban population as well as ageing population. Since early 1990s, acknowledging the importance of the healthcare sector, the Vietnamese government formulated various policies to promote the development of the domestic health care sector in general and of the pharmaceutical industry in particular. Although the effectiveness of such policies is still controversial, the development of the health care sector is quite remarkable. The World Bank frequently hailed Vietnam for achieving health outcomes better than what its income level would suggest [5]. 

However, as of 2015, it is estimated that 90% of national drug expenditure depends on imported sources [3]. Looking at the trade patterns in recent years, although the pharmaceutical industry has performed significantly well, doubling the volume of exports in the interval 2011–2015, it seems that Vietnam still lags behind other neighboring economies in the East Asian region. In 2015 Vietnam has exported pharmaceutical products for a value of 113 million USD, equal to 4.6% of its total imports in pharmaceuticals, which is considerably lower with respect to other regional competitors such as China (6.9 billion USD exports, equivalent to 36% of its total import), Indonesia (586 million USD exports, equivalent to 79% of its total import) and Thailand (438 million USD, equivalent to 21% of its total import). Moreover, the country exhibits increasing trade deficits with both East Asian mature and emerging markets, such as Japan, South Korea, China, Malaysia and Thailand [6], thus revealing a dependent position of the country in the international division of labor and the lack of competitiveness of its domestic production. Indeed, in most cases local manufacturers only manage the lowest stages of drug production, mainly importing raw materials, and are far from self-reliant in producing raw medicinal materials and inventing new drugs [7]. 

So far, the literature on the Vietnam pharmaceutical industry is quite limited. Apart from periodical reports from analytical firms such as BMI, FPT Securities, VietinBank, certifying an increasing interest by the private investors towards the sector, few updated studies attempted to investigate the development of pharmaceutical industry with a comprehensive approach. In this regard, in the last fifteen years the academic literature on the topic has been revealed to be quite fragmented, focusing on wider issues (such as the structure of the country’s healthcare system) or stressing specific sectorial topics. For example, Ladinsky, et al. [8] analyzed the evolution of the structure and the funding of the Vietnamese healthcare system providing some brief arguments regarding the pharmaceutical sector. On the other hand, Simonet [9] reviews the transition policies implemented in the 90s and highlights their impact on healthcare provision and in terms of modernization of the pharmaceutical industry. 

Following the WTO accession, a stream of research has investigated Foreign Direct Investment (FDI) entry strategies in the Vietnamese pharmaceutical market trying to assess the main determinants in terms of localization and entry mode and stressing the effects of regulations, licensing and entry barriers on market expansion [10,11]. In parallel, some other contributions explore the macroeconomic constraints that are likely to affect the performances in the sector. From this perspective, Nguyen and Roughead [12] investigate the pricing policies for pharmaceutical products and point out the irrational regulations responsible for the distortion of drug price in the market. However, as stressed by other studies, it seems that the slow development of the pharmaceutical industry is ascribable to further contextual factors other than pricing policies [13,14]. In addition, the recent settlement of the European-Vietnam Free Trade Agreement (EVFTA) has stimulated a series of contributions intended at assessing the impact of trade openness and tariff reductions on the Vietnamese pharmaceutical enterprises’ performance [15] and quantifying the country’s potential gains in terms of pharmaceutical imports [16].

Acknowledging the contribution of previous studies, this paper aims to provide a comprehensive analytical discussion regarding to the development of the pharmaceutical industry in Vietnam. The main objective of the paper is to analyze the evolution of the pharmaceutical industry development by taking into account all the policies put in place by the government and remarking on some difficulties in the implementation and some structural weaknesses. 

The paper is structured as follows. Section 2 focuses on the development of Vietnamese healthcare market in general and on pharmaceutical products in particular on the demand side, its main trends in term of consumptions and import flows. Section 3 describes the evolution of Vietnamese pharmaceutical industry on the supply side and related key state policies. A comprehensive analysis of the major problems hindering the development of the industry is presented in Section 4. The final section concludes the research with some recommendations.

## 2. The Development of the Health Care Market 

According to multiple reports, Vietnam is a fast growing market for pharmaceutical products that is very attractive to foreign firms at present and in the near future [2,17,18]. The market’s growth rate averaged 17–20% during 2010–2015, making it one of the fastest markets in the region [19].

**Figure 1:**
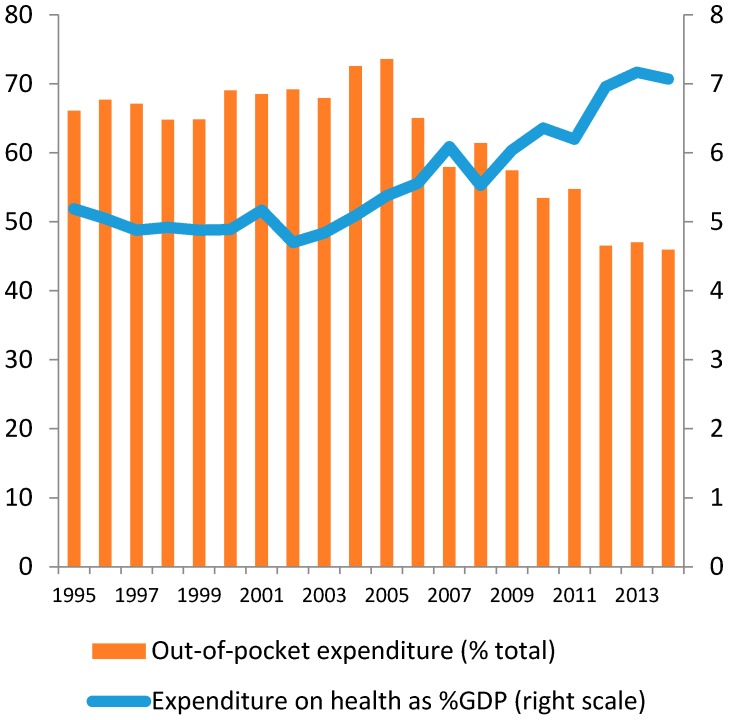
Expenditure on health as %GDP and out-of-pocket expenditure.

Total expenditure on health accounted for 5% of GDP in 1990s, increasing to over 7% in 2014, which is higher than almost all other countries in developing Asia [5]. At the same time, out-of-pocket expenditure reduced from around 70% (1990) to 45% (2014) (see Figure 1).

**Figure 2:**
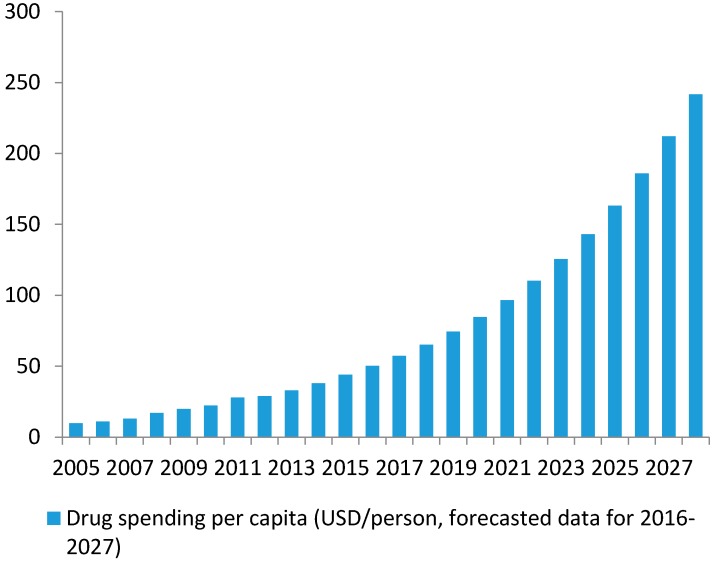
Drug spending per capita in Vietnam.

Growth in drug spending per capita has been continuous and stable. The average drug expenditure per capita was 9.85 USD in 2005, 22.25 USD in 2010 and up to 44 USD in 2015 [20]. The average growth rate reached 17.7% per year between 2005 and 2010 and 14.6% between 2010 and 2015. Supposing that the expenditure maintains a growth rate of at least 14%/year up to 2025, it is estimated that drug spending per capita will double to 85 USD in 2020 and quadruple to 163 USD in 2025 (see Figure 2). 

The rapid growth of pharmaceutical demand in Vietnam can be attributed to a series of economic and social trends. Among these, we identify three key drivers including: (i) high economic growth and increasing personal income; (ii) rising population and rapid urbanization; and (iii) rapid ageing, which all contribute to people’s higher living standards, stimulating the aggregate consumption of pharmaceutical products. On the other hand, several negative effects such as pollution, insufficient food safety or unsafe living and working conditions also exert impacts on the demand as well. In this regards, such dynamics look similar to other emerging countries such as China, where higher longevity, combined with environmental and pollution problems, are increasing chronic diseases like respiratory illnesses, cancer, diabetes, as well as obesity [21].

(1) High economic growth and enhanced personal income: For the past 20 years, Vietnam’s economy rapidly expanded at an average yearly rate of 7%. In the same time, GDP per capita increased from 288 USD in 1995 to 2110 USD in 2015 [22], equivalent to growth of over 10% per year in nominal terms. Strong income growth and increasing health awareness are favorable conditions to increase expenditure in healthcare in general and in pharmaceutical products in particular [23,24].

(2) Rising population and rapid urbanization: During the last two decades, Vietnam has experienced a dramatic increase in the population, passing from 72 million people in 1995 to 91 million in 2015 [25] and resulting in a consistent expansion in the domestic market size. At the same time, urbanization triggered a spatial and demographic expansion. According to the World Bank, urban areas in Vietnam have developed spatially at 2.8% per year, which is among the fastest rates in the Asian region World Bank [26]. As of 2015, the urban population is about 33.5% and it is expected to reach 40% by 2020 and 50% by 2030 [27]. In these contexts, the emergence of new urban middle-classes is likely to be associated with changes in people’s behaviors and consumption patterns positively affecting the demand for healthcare and pharmaceutical products [28,29]. 

Moreover, the mass migration from rural areas leads to a concentration of healthcare services in the two biggest cities of the country, Hanoi and Ho Chi Minh City. Such a dynamic makes urban hospitals seriously suffering from ongoing undercapacity. It is estimated that while Hanoi and Ho Chi Minh City account for less than 20% of Vietnam’s population, their hospitals need to serve 60% of all patients in the country [30].

The unsatisfied demand of public healthcare services has encouraged the activities of private companies providing additional channels for domestic consumption of pharmaceutical products [18]. At the same time, urban life also generates negative externalities on the life standards and health conditions [31] stimulating an average increase in the personal consumption of drugs.

**Figure 3:**
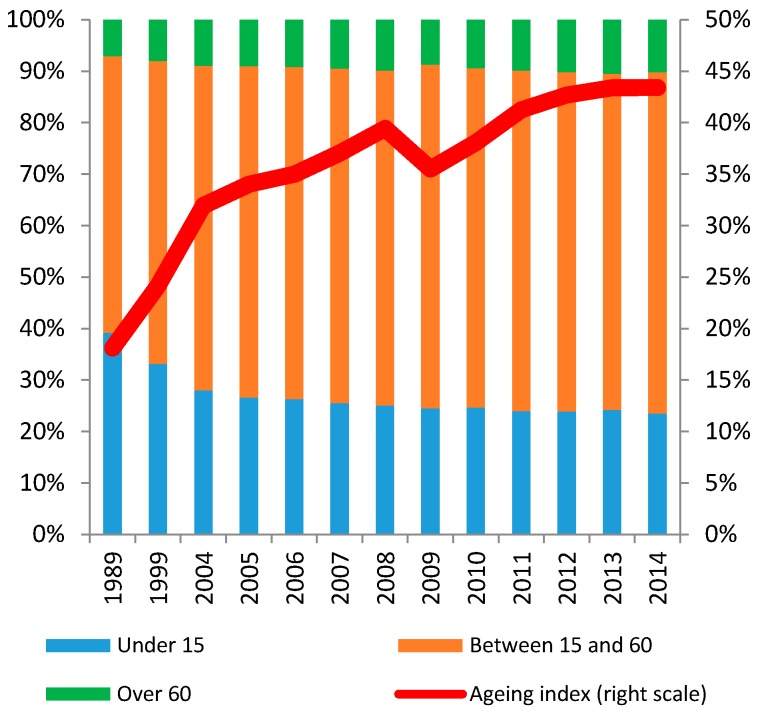
Population structure and ageing index.

(3) Rapid ageing: According to the Ministry of Health, Vietnam has gradually experienced the initial stage of ageing process [20]. The number of people above the age of 60 reached 10.2% in 2014 in comparison to 7.1% in 1989. During the same time, the number of people under the age of 15 quickly decreased from 39.2% to 23.5%. As a result, the ageing index, i.e., the ratio between the number of people over 60 years old and the population under 15 years old, increased from 18.2% (1989) to 43.3% (2014) (Figure 3). According to the United Nations, by 2040, the number of people older than 65 is projected to almost triple to 18.4 million, and to account for 17% of the population [32]. As population currently increases at around 1% per annum, the speed of ageing in Vietnam is among the fastest seen globally to date [33].

The improvement in life expectancy, however, also makes Vietnamese people more likely to suffer from chronic diseases related to old age. A similar trend triggers the demand for pharmaceutical products but, on the other hand, represents a serious risk for the sustainability of the Vietnamese healthcare system.

While ageing is not the sole cause, it is a significant contributing factor to the explosion of non-communicable diseases (NCD) in Vietnam in recent years [33]. According to the Ministry of Health, in 2010 NCD accounted for over 70% of the causes of hospitalization in Vietnam while the remaining 30% refers to infectious diseases, accidents, injuries and poisoning [34].

Moreover, it is estimated that the share of older people having at least one difficulty in activities of daily living increases from around 28% among those 60–69 to over 50% for those 80 and older, with somewhat higher incidence of difficulties among women [33]. In addition, according to the Ministry of Health (hereinafter MoH), healthcare expenditure for elder people on average is 8 times higher than for young people [35].

Based on this, it appears clear that the process of population ageing requires higher health spending and a more productive allocation of the public funds and resources. This also interests the pharmaceutical sector as older people typically consume more drugs, and thus inefficient pharmaceutical practices become increasingly expansive as their direct recipients age.

**Figure 4:**
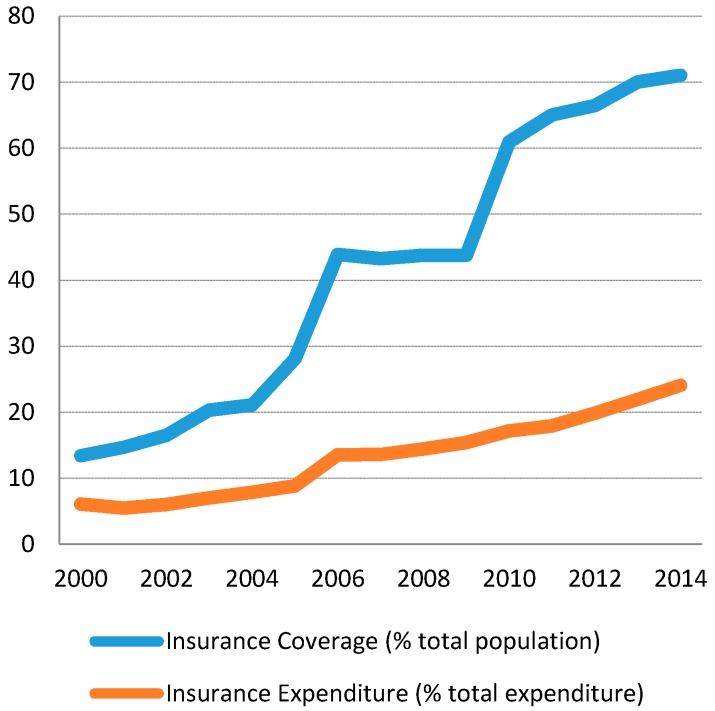
Insurance coverage and insurance expenditure.

In this regard, Vietnam has undertaken the decision to develop a health financing system based on social health insurance, which aspires to achieve universal health coverage. The Law on Health Insurance came into force in July 2009, aiming to provide insurance coverage to 100% population by 2018 [36]. Following this principle, the 2013 Constitution of Vietnam stipulates that: “The State and society invest in development of the protection and care of the people’s health and implement universal health insurance”. Nevertheless, the health insurance scheme has not turned out to be so proficient, and this is partially due to a big gap between the insurance coverage as a share of total population and the effective share of insurance expenditure out of the total expenditure. This gap widened over time during the interval 2000–2014 (see Figure 4). So considering 2014, for example, though the insurance coverage reaches more than 70% of the population, the insurance expenditure only accounts for 24% of total expenditure on health. An interpretation of this feature may be linked to the consumption behaviors of the middle- and upper-class patients who are increasingly oriented towards purchasing drugs from private pharmacies, which are not connected to the public service delivery or health insurance system [5]. In addition to this, there are other distortions connected to the management and the governance of the social health insurance system, whose inefficient organization, territorial fragmentation and administrative dysfunctionality often give rise to unnecessary transactions costs [37].

## 3. Evolution of Pharmaceutical Industry

### 3.1. Overview of the Industry

#### 3.1.1. State of Development of the Domestic Production

According to some reports, the pharmaceutical industry in Vietnam has reached an intermediate level of international integration resulting in the development of a domestic pharmaceutical industry that is specialized in manufacturing generics and exporting non sophisticated pharmaceutical products to other countries [3]. However, it should be noted that so far the domestic industry mainly produces drugs out of imported raw materials while the value-added of exports is very small in comparison to the inputs imported for production. 

The value of drugs produced in Vietnam accounted for 0.72% of GDP in 2014 and only 2.18% of national industrial revenues [20]. It is forecasted that the pharmaceutical industry will expand at 15.5% in the next 5 years, contributing to 2.2% GDP in 2017 [2].

In 2015, the domestic industry supplied up to 50% of the domestic demand while imports covered the remaining half, not yet counting values of input materials and active ingredients [20]. In this regards, approximately 60% of pharmaceutical end products, 90% of active pharmaceutical ingredients, and most raw materials for the production of pharmaceuticals are currently imported. On the other side, foreign enterprises are responsible for an estimated 20% of the domestic pharmaceutical production [38].

In 2015, the distribution system consisted of 1200 companies (both domestic and foreign manufacturers); 1180 public health hospitals; 170 private hospitals (mainly located in the big cities) and 54,250 retail drug stores [17]. Foreign Direct Investment logistic companies and foreign pharmaceutical companies are not permitted to distribute pharmaceutical products directly in Vietnam. 

#### 3.1.2. Imports and Exports

Focusing on the evolution of the sectorial trade flow, the total pharmaceutical imports reached 1.8 billion USD in 2013, after an average growth of about 18% during the interval 2008–2013 [19]. On the other side, the export volumes still appear to be small since domestic firms can only produce conventional drugs for which international supply is abundant and highly competitive.

**Figure 5:**
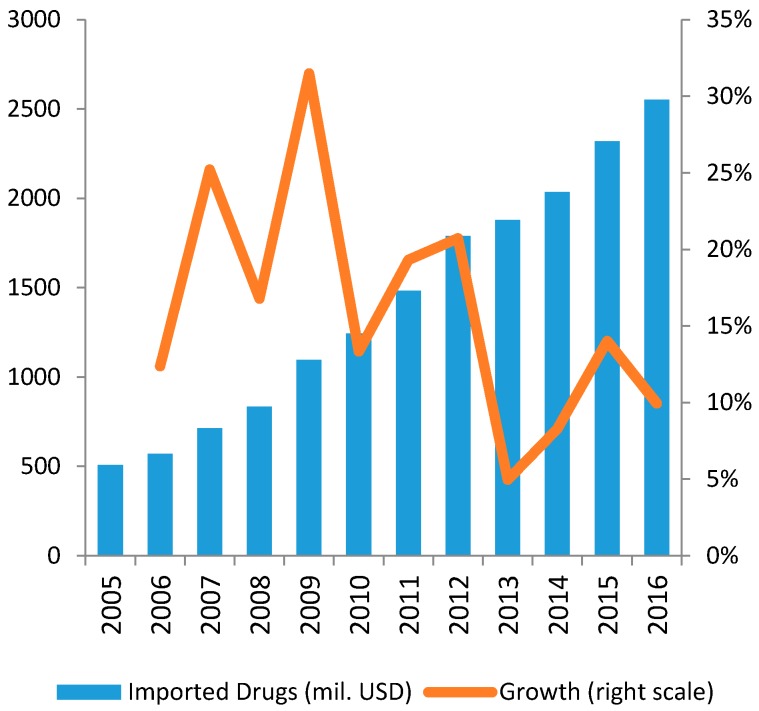
Import Volume of Drugs in 2005–2016.

**Figure 6:**
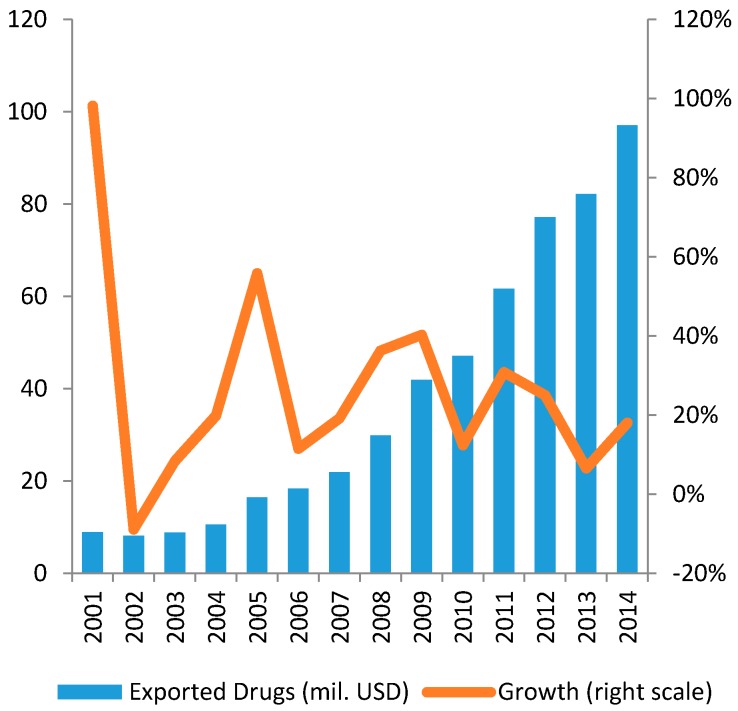
Exports Volume of Drugs in 2001–2014.

Exports of Vietnamese pharmaceutical products are still low in value (about 100 million USD in 2014) but show a remarkable growing trend (see Figure 5 and Figure 6). 

In this context, Vietnamese drug manufacturers seem to target less-advanced and competitive markets to benefit from low barriers to entry. South East Asian countries such as Laos and Cambodia are preferred targets, while African states are growing in terms of market quotas. Meanwhile, the Middle East and the ex-Commonwealth of Independent States (CIS) are both being considered as potential future customers for Vietnamese-made pharmaceuticals [39].

Based on these trends, Vietnam seems to assume a dependent position in the international division of labor as it basically imports raw materials and high value-added inputs from internationally competitive regional players, such as Japan, South Korea, Malaysia, Thailand and China and exports low value-added pharmaceuticals to less advanced markets (according to UN Comtrade Database 2017). This dynamic results in increasing sectorial deficits that are likely to expand further given the lacking capability of the internal production to address the domestic demand. 

#### 3.1.3. Agents, Dynamics and Governance in the Vietnamese Pharmaceutical Sector 

Similar to other sectors, the pharmaceutical industry in Vietnam is structured in a mix of both public, private and foreign agents. In 2015, the industry counted 170 firms, including 20 joint-venture foreign-invested firms, with the largest one controlling less than 5% of the market [5]. To operate in the market, local manufacturers have to comply with the code of Good Manufacturing Practice (GMP), importers with Good Storage Practice (GSP), distributors with Good Storage Practice (GSP) and Good Distribution Practice (GDP) and retailers with Good Pharmacy Practice (GPP) (see Table 1). 

Although the largest former State-Owned Enterprises (SOEs) have been equitized, they continue to prosper on the basis of close relationships with distributors and hospitals in their areas. Procurement is largely managed by individual hospitals, and bidding is open to corruption, with allegations of high markups for producers making payments to hospital administrators [5]. Most local pharmaceutical manufacturers comprise small-scale operations with outdated manufacturing technology and duplicated production processes. As a result of this, local producers massively rely on imports. For example, in 2013 the total turnover of the 9 biggest domestic pharmaceutical firms was about 450 million USD, equal to 25% of total imported drugs (see Table 2). 

Foreign pharmaceutical companies are not allowed to directly distribute on the market, so they must sell their products to domestic pharmaceutical distributors. Typically, foreign investors enter the domestic pharmaceutical market by establishing a joint-venture company with a local partner or as a wholly foreign-owned enterprise (WFOE) to import and sell their pharmaceutical products to a licensed domestic distributor in Vietnam [10].

At the same time, some foreign-owned companies active in the wholesale sector have managed to control a vast segment of the market, particularly regarding the imported drugs. Through their large brand portfolios, they have become key players in the supply-chain (Diethelm Vietnam, Zuellig Pharma Vietnam, Mega Lifesciences).

Product registration is under the responsibility of the Drug Administration of Vietnam and requires long local trials as it is conducted case by case, with the regulator retaining considerable discretion. Under these conditions, genuinely private and foreign firms are disadvantaged in market access, enabling small producers of generic drugs to survive in what on the surface looks like a highly competitive market [5].

Before being sold in Vietnam, a drug must be registered to the MoH with a declared price set by the registrant company. The Ministry issues a marketing authorization, usually valid for 5 years, after which the product must be re-registered. The MoH can allow medicines without a registration number to be marketed on a case-by-case basis, to avoid shortage of medicines. By law, within 6 months from the date of receipt of a complete and legitimate registration applications, the MoH shall issue medicine-marketing authorization for the medicine.

Newly developed drugs may take around 5 years to enter Vietnam; this includes clinical trials of 2.5 years, and then the same time for the approval procedure. In principle, existing drug registration takes maximum 6 months, and is valid for 3–5 years. In practice, it can be anything between 12 and 24 months. The firms also have to reapply for the license at least 6 months prior to the registration expiration date. Yet, the process of renewal can take up to 8–12 months.

### 3.2. The Evolution of Government Policies in the Pharmaceutical Industry

In Vietnam, the key central government agency responsible for healthcare issues in general, and pharmaceutical management in particular, is the Ministry of Health (MoH). The MoH’s basic functions, authority and responsibilities are set forth in the government’s State Decree No. 15/CP dated 3 February 1993. In 1996, The Drug Administration of Vietnam (DVA) was specially established with the responsibility for state management of pharmaceuticals. Since then, DVA acted as the pharmaceutical regulatory authority under the supervision of MoH.

In general, the ultimate goal for the development of pharmaceutical industry in Vietnam is to “fully meeting the domestic demand” [40], reflecting the role of an essential sector rather than of a profit-seeking industry. To achieve this, the government together with MoH and DVA has managed short-term policies based on long-term development strategy up to next 10–20 years. For example, the National Drug Policy issued in 1996 is a development strategy up to 2000 with the outlook to 2010 while the current development strategy (issued in 2015) is up to 2020 with the outlook to 2035.

Investigating the government planning strategies related to the sector, it is possible to identify three main development periods of Vietnam’s pharmaceutical industry from 1990 to 2017.

#### 3.2.1. Transition Period (1990–2005)

Prior to 1990, the pharmaceutical industry was under-developed and dominated by state-run companies with low production capacity. Average drug expenditure per capita was just about 0.5–1 USD per year and the quality of drugs was not of great concern [3]. The economic reform process known as “Doi Moi”, initiated in 1986, led to important policy shifts in the health care system in the early 1990s. 

First, several market-oriented measures were implemented, such as the introduction of user fees at public health facilities, the legalization of private pharmacy and medical practices, and the liberalization of the production and sale of pharmaceuticals. Free access to health care was gradually replaced by a system of direct payment by patients [41]. The implementation of Central Resolution 4 (dated 14 January 1993) and Decision 58/QD-TTG (dated 3 February 1994) supported the opening of pharmaceutical industry to the private sector which led to an explosive growth of drugstores and pharmaceutical companies with increasing variety of drugs. It is estimated that pharmaceutical production increased by 300% while importation of pharmaceutical products raised tenfold between 1988 and 1992 [42].

The provision of free medicines dispensed through the public health system was also discontinued [41]. As a result, Vietnam’s near universal, publicly funded and provided health services were converted into an unregulated public–private mix [43].

In 1996, the Government issued the Resolution No. 37/CP (dated 20 June 1996), approving two influential and important policy milestones affecting the pharmaceutical sector, namely: (1) Strategic orientation on people’s health care to 2000 and vision to 2020; and (2) the Vietnam National Drug Policy. The Resolution was issued to improve the coordination of pharmaceutical policies with two basic goals: (1) ensuring regular and adequate supply of good quality medicines at affordable prices; (2) and ensuring a rational use of medicines. The Resolution 37 was strongly credited for its initial crucial support to the development of pharmaceutical industry substantially in all aspects of state management of pharmaceuticals including manufacturing and supply of drugs, quality assurance and safe and rational use of drugs [20].

However, the negative side of the Resolution 37 is that the rapid shift to free market regulation made medicine prices unaffordable for many people. In 2005, after adjusting for Purchasing Power Parity, the prices for innovator brands and lowest-priced generic equivalents in the public sector were 46.58 and 11.41 times the international reference price respectively [44]. 

Acknowledging the weaknesses of such legislation and regulations, MoH began drafting the first Pharmaceutical Law in 1997, which was finally enacted in 2005 after almost a decade of consultation, discussion, drafting and development. The new law provided a comprehensive legislative framework for all aspects of the pharmaceutical sector, including specific medicine pricing provisions [44]. 

Overall, between 1990 and 2005, the pharmaceutical industry has partially undertaken a transition from state monopoly to a competitive market mechanism attracting public capital and foreign direct investments. In addition, the industry regulations gradually started to address the requests of the private sector allowing private pharmaceutical companies to be involved in import and export of medicines (subject to eligibility criteria in terms of capital, manpower, facilities, technology and customer relationship). 

#### 3.2.2. Integration Period (2005–2017)

With the new Pharmacy Law passed in 2005, the government set out a master plan for the long-term development of the pharmaceutical industry [45]. The ultimate goal was to transform the pharmaceutical industry into a “spearhead techno-economic branch” ([45], Article 3.1) by building a network of circulation, distribution and supply of pharmaceutical products, thus “ensuring sufficient pharmaceutical products to meet the people’s demands” ([45], Article 3.5). In this context, the government encourages organizations and individuals (whether Vietnamese or foreigners) to “conduct scientific research into preparation technologies and biotechnologies for manufacturing new pharmaceutical products; to invest in production of pharmaceutical products” ([45], Article 3.2). The government also commits to “protect lawful rights and interests of organizations and individuals in pharmacy research, trading and use of pharmaceutical products in Vietnam” ([45], Article 3.6).

The government aimed to reach 60% of pharmaceutical needs met by local manufacturers by 2015. However, by 2015, the target was not reached due to two main obstacles: (1) lack of capacity of the local industry in supplying key raw materials and (2) lack of adequate human resources. While the investment in R&D of private sector is very low, that of SOEs is also far from reaching their targets.

The accession to WTO in 2007 opened the domestic economy to foreign companies, which strongly promoted competitive dynamics in the market. Vietnam committed to reduce tariffs on 47 pharmaceutical products from 10–15% in 2006 to 2.5% in 2012 [46]. According to Vietnam’s WTO commitments, foreign-invested companies and their branches have been allowed to import drugs directly into Vietnam since 1 January 2009, but still not allowed to distribute them to the end-user. In parallel, most of the limitations in market access for foreign investors in the hospital, medical and dental services in cross border supply and consumption abroad were removed. Vietnam has agreed, as part of its WTO accession commitments, to extend trading rights (the right to import and export independent of government-approved channels) to pharmaceuticals since 2009 as well. These trading rights have further legal foundation in regulations relating to import and export rights such as Decree 23/2007/ND-CP (dated 12 February 2007) and Decision 10/2007/QD-BTM (dated 21 May 2007) issued by the Ministry of Industry and Trade.

Since 2007, domestic drug companies have been required to achieve the standards equivalent to GMP—ASEAN, and subsequently, to GMP—WHO, PIC/S and EU—GMP, etc. This policy was motivated by the necessity of domestic pharmaceutical industry to reach the required standard to integrate into international market. As a result, the number of enterprises being certified by the Drug Administration of Vietnam (DAV) for reaching GMP—WHO standards has progressively increased. In 2015, there were 150 domestic manufacturers reaching WHO—GMP standard [1]. 

In 2010, another key milestone of government policies regarding the pharmaceutical industry was the Five-year Health Sector Plan 2011–2015. The main objective set out in the Plan was to “develop the domestic pharmaceutical industry, increase efficiency in management and use of drugs and medical biological products” [47]. In order to implement this objective, the Plan set out some focal tasks to be implemented by the pharmaceutical sector, which are summarized in the following four groups [20]: (1) Improving policies and regulations related to the pharmaceutical industry; (2) Increasing access to medicines; (3) Managing drug quality; and (iv) promoting a safe and rational use of drugs. To support the implementation of the Plan, the government issued two documents in 2013 and 2014, respectively: (1) Prime Ministerial Decision No. 1976/QD-TTg (2013) about medicinal ingredient development up to 2020 and orientation to 2030; and (2) Prime Ministerial Decision No.68/QD-TTg (2014) proposes the National Strategy for Development of the Vietnamese pharmaceutical industry to the year 2020 and vision to 2030.

As of 2016, the Plan was generally evaluated to be successful as most targets were achieved before the deadline [34]. In particular, the access to medicines has continued to be strengthened (reaching an average density of 2123 people per retail pharmaceutical outlet) [20]. In June 2015, WHO certified that Vietnam has a fully equipped national regulatory system that ensures the safety and efficacy of vaccines produced and used in Vietnam. This represents a necessary condition for Vietnamese-produced vaccines to participate in supplying drugs funded by international organizations, but it also constitutes a good starting point to upgrade the domestic production and to increase the international sales [20].

#### 3.2.3. New Expansion Period (2017–Onward)

In June 2016, the National Assembly promulgated the new Law on Pharmacy, which came into force since 1 January 2017, replacing the old version passed in 2005. This reform is combined to the promotion of a “Masterplan of Medicinal Ingredient Development to 2020” and the “National Strategy for Development of the Vietnamese Pharmaceutical Industry to 2020”. These policies are likely to mark a consistent shift in the government industrial policy approach towards the pharmaceutical sector which appears to have been targeted as a strategic sector, given its potential in terms of market expansion and knowledge spillovers.

In comparison with the previous 2005 Law, the 2017 Law adds four new chapters on:

1. State policies on pharmacy and pharmaceutical industry development;
2. Pharmacy practice
3. Clinical pharmacy; and
4. Management of drug prices

The 2017 Law introduces new supports to the investments in drug production, drug materials, essential drugs, social diseases drugs, and vaccines. The government provides new incentives to R&D in production technology and biotechnology to manufacture new drugs. In detail, the Law establishes prioritized fields of development in the pharmaceutical industry, including: (1) Research and production of drug materials from medicinal material sources available in Vietnam to serve the preparation and production of medicinal material drugs and traditional drugs; (2) Manufacturing of drugs upon the expiration of patents and relevant protection titles, and of vaccines, biological products, medicinal materials, drugs from medicinal materials, traditional drugs and rare drugs; and (3) Development of medicinal material sources and medicinal material culture and cultivation zones; conservation of gene sources and development of precious, rare and endemic medicinal material species and varieties.

The changes introduced to the Law on Pharmacy are expected to significantly affect the development of the domestic industry. So far, the domestic firms have dominated the generics market while foreign enterprises controlled the market share for non-generic high quality drugs. The new Law aims to change such landscape by prioritizing the purchase of domestically produced products, including domestically manufactured generics and biosimilar, herbal and traditional medicines manufactured from domestic herbal ingredients, and drugs manufactured in domestic facilities satisfying good manufacturing practice standards

On the other hand, the new Law also favors the establishment of foreign investment enterprises (FIEs) in the pharmaceutical sector since it is necessary for accessing the most innovative drugs and techniques. Another aspect to highlight is the removal of Five-Year Rule for Clinical Trials. In the old Law, a new drug could be circulated in Vietnam only if it had already been adopted in its country of origin for at least five years; otherwise, it needed to undergo clinical trials for registration purposes. On the other side, the new regulation facilitates the access to new drugs, especially those related to life-threatening diseases [48].

## 4. Analysis of Major Challenges in the Vietnamese Pharmaceutical Industry

In the previous sections, the analysis of domestic demand and supply of pharmaceutical products in Vietnam highlights some elements of problematic development. While the demand side exhibits a significant potential for sustained growth, the trends in the domestic industry seem to be characterized by a slower development path due to a series of inefficiencies. The next section will identify the major problems that are currently hindering the development of domestic pharmaceutical industry. 

### 4.1. No Long Term Strategy

The Vietnamese Government has promulgated a series of policy documents guiding the development of the national pharmaceutical industry for the period with a vision toward 2030. However, Vietnam has not yet had a particular master plan dedicated to the development of the pharmaceutical industry in long-term. A sectorial master plan might represent an important source to guide investors, especially the domestic private investors, foreign investors as well as local existing pharmaceutical companies in planning new development, expansion and upgrading of production facilities, in enhancing their competitiveness and mitigating the risks of market-failures. In brief, a master plan might define the direction and priority of the development areas, the modern level and status the pharmaceutical industry needs to reach at the end of this decade, the dispose of reasonable investment on geographic area, level of quality requirement for local pharmaceutical products. In a similar perspective, the plan needs to focus on priority areas to develop and modernize in this decade. The identification of the priority areas is extremely important to address the implementation efforts. 

### 4.2. Low Value Added Production

Pharmaceuticals produced in Vietnam face difficulties in competing on international markets because they mainly consist in drugs for treating common diseases or only generic drugs that have not yet achieved bioequivalence standards, thus not suitable for exports [20]. Domestic manufacturers have not adequately invested to ensure bioequivalence, bioavailability and therapy equivalence of medicines—proving the same quality of generics as origin brands. Conversely, they have only specialized in the manufacturing of pharmaceutical and chemical equivalents of pharmaceutical products. In this regards, about 1000 out of 2000 drug substances registered in Vietnam belong to foreign firms, while 500 of them are owned by domestic companies, mainly focusing on antipyretics, pain relievers, vitamins and supplements [49].

Domestic manufacturers are struggling to meet international standards, such as PIC/S–GMP, EU–GMP, aiming to produce high quality drugs in order to be more likely to win a contract for ETC (Ethical Channel) and thus expand to export markets [3]. Therefore, local producers are mainly specializing in common or generic drugs’ production, being less keen on investment in R&D for producing products with high knowledge content, technology and know-how in order to enhance value, quality and effectiveness of drug [49].

### 4.3. Dependence of External Material Inputs

**Figure 7:**
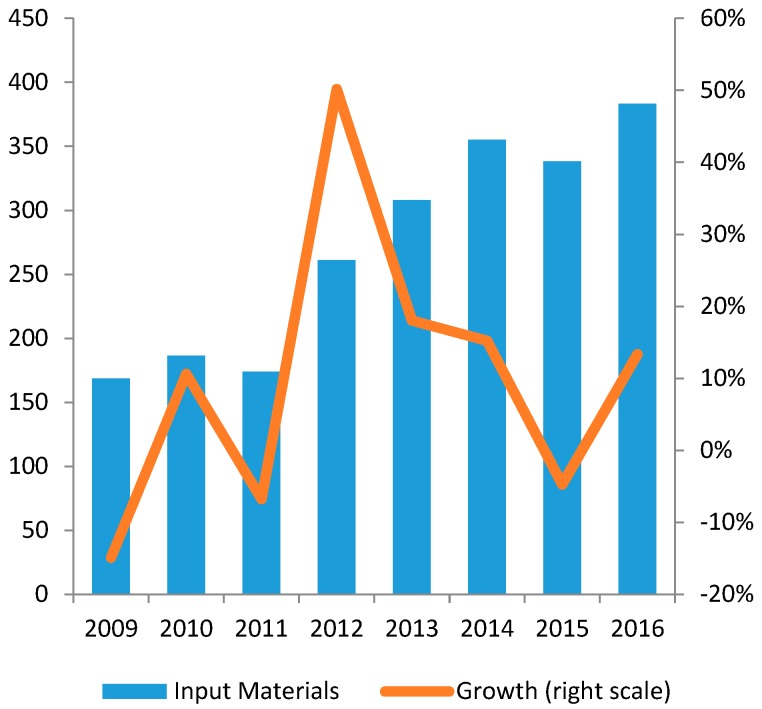
Import Volume of Input Materials in 2009–2016 (mil. USD).

**Figure 8:**
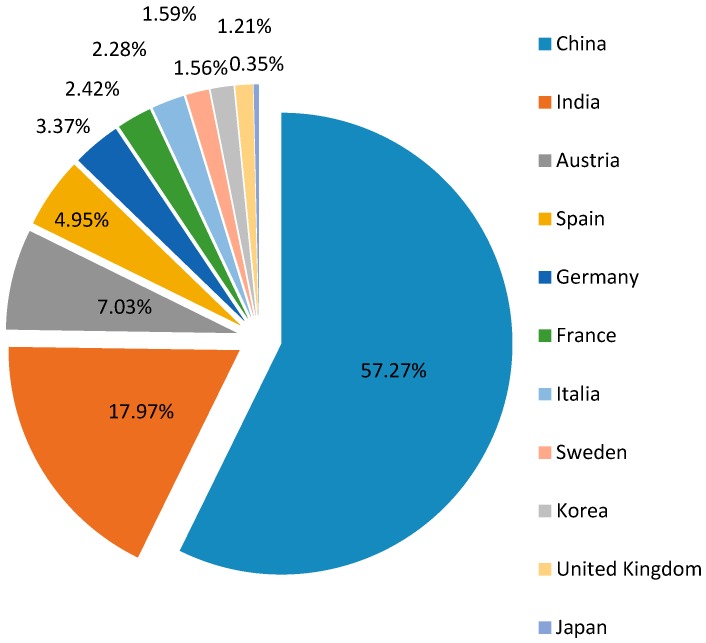
Biggest import partners in 2013.

Vietnam needs to import the majority of input materials for pharmaceutical production (see Figure 7). The biggest import partners are China and India, respectively accounting for 57% and 18% of total import value in 2013 (see Figure 8). The dependence on imported materials makes the industry vulnerable to external risks such as exchange rate fluctuations or supply shocks [46]. Furthermore, due to high input material cost, according to MoH, the average export price of Vietnam drugs during 2011–2013 was about 20–25% higher in comparison to India and China [18]. From this perspective, the lack of local-value added, as well as the limited R&D facilities, insufficient financial capacity and poor management contribute to the low competitiveness of the domestic products on international markets [13,46].

In detail, nearly 95% of imported active pharmaceutical ingredients are antibiotics, vitamins, antipyretic, analgesics and anti-spasmodic drugs [12], reflecting a concentration of domestic pharmaceutical production on only some therapeutic classes. As mentioned above, this can be attributed to the fact that most companies in the industry are small and lack of adequate investment (see Section 3.1.3).

### 4.4. Distorted Distribution Network

The current distribution system of pharmaceutical products is considered fragmented, inefficient and with poor transparency [12]. In 2015, there were approximately 57,000 pharmacies in Vietnam, corresponding to about 6.6 outlets per 10,000 people, the ratio of which is higher in large cities than in rural areas. For instance, according to figures released by the MoH, the number of pharmacy outlets to 10,000 residents in Ho Chi Minh City (HCMC) was around 45 in 2010, far greater than the national average figure [18].

So far, the conditions for the establishment of a pharmacy have not been strictly restrictive [1]. The uncontrolled involvement of small local distributors and the lack of a clear legislative guidance increase the inefficiency of the market and raise the final price of drugs [12].

In addition, according to MoH, the fact that the range of imported products is wider than those locally produced generates trading duplication of some active substances [20] and there are no proper barriers to limit the registration of overlapping products [49].

### 4.5. Price Distortion

Vietnam’s current pharmaceutical procurement system is highly decentralized and complex. Hospitals in Vietnam mostly purchase pharmaceuticals through bidding, which is subject to a price ceiling per medicament set by the regional health department. Those ceilings might greatly differ between areas, resulting in wide differentials in prices of medicines across facilities and regions of the country. Sale of drugs is still the major source of income for the State health care system, therefore some provinces have decided against service charges but they use the sale of pharmaceuticals as their primary source of income [8]. There are also irregularities in the procurement processes [33]. As a result, local people tend to pay a higher cost for medication. A study found that the prices of winning bids were 130% to 245% higher than prices of imported drugs [37].

Control of drug prices is still a considerable challenge due to the lack of specific mandated authorities and responsibilities between related ministries and sectors. There is no unified view of the mechanism for drug prices management. A tendency to consider drugs as equal to other consumer goods leads to neglect the dual character of pharmaceuticals, which clearly is both economic and social. This does not bring a consensus on the drug price control mechanisms that conforms to a market economy [49]. The Pharmaceutical Law assigns to the MoH the function to take the lead on drug price management, but it does not clearly assign responsibilities to other sectors, nor set up a multi-sectoral committee or commission for drug price management, leading to substantial difficulties in the implementation and inabilities to ensure the transparency and openness in managing drug price since multi-sectoral action is required.

The Pharmaceutical Law affirms that declaration and re-declaration of drug prices must “ensure that drug prices are no higher than drug prices of countries in the region with similar health and economic conditions as Vietnam” ([45], Article 36.1). However, so far the Ministry has not yet issued this list because of difficulties in determining the countries having similar health and economic conditions [20].

### 4.6. Ineffective Quality Control and Supervision

In its latest report, the MoH had to even admit the “irrational use of drugs remains popular and over-the-counter sale of drug is prevalent while safe use of drug remains a concern of health facilities” [50]. This is of particular concern for traditional drugs as “inspection, check and control of origin and quality of pharmaceutical materials used for production of traditional medicines, drugs made of herbal materials and the use of these drugs in traditional medicine facilities are not done properly” [50]. 

For western drugs, the issue is less severe but quite worrying. Indeed, the proportion of antibiotics used is often higher than required, with rising risks of antimicrobial resistance. Health workers still have the habit and tendency to prefer prescribing and using strong and more expensive imported drugs to meet healthcare needs [20]. Generally, Vietnamese people have less confidence in domestic pharmaceutical products and even domestic companies occasionally failed to provide sufficient evidence of product efficacy in comparison with imported drugs [1]. This not only affects the quality control system but also strongly influences the development of the domestic pharmaceutical industry and increases treatment costs.

One of the key reasons for such an ineffective quality control is that the state budget funding for the domestic drug quality assurance system remains low, while the number of new drugs in the market increases quickly. The number of new drugs produced and circulated in the market continues to grow, including products with new modes of administration, new active ingredients, bio-pharmaceuticals, high-tech drugs (nanosomes, liposomes). Although the MoH and its agencies seem quite active in performing quality control system (see Table 3), the limited state budget certainly results in the deficiency of equipment and standard materials for effective operation [20].

## 5. Conclusions

Vietnam has a potential market for pharmaceutical products, which is expected to expand rapidly in the near future. However, the domestic market is currently dominated by foreign products because of the under-development of domestic firms’ tangible and intangible assets. This may cause significant losses to Vietnam on the international market and in trade agreements. 

On the other hand, Vietnam cannot exclusively rely on the cheap labor cost advantage, as this does not represent a significant competitive factor in the pharmaceutical industry. Indeed, the pharmaceutical sector is a high-tech industry in which the main drivers of product competitiveness are linked to research and development activity, technological endowments, utilization of modern manufacturing processes, manufacturing process automation and total quality management system. 

Based on this consideration, the Vietnamese pharmaceutical industry still seems to be in its early stage of growth, though the overall share of GDP spent on health in Vietnam is relatively high compared to other countries. This suggests that further reforms of health financing need to focus on improving efficiency rather than mobilizing additional resources for health, and enacting policies that more effectively reduce the financial burden on households using healthcare products and services.

Based on the findings in prior sections, this study proposes some recommendations to improve Vietnam’s pharmaceutical industry participation in the global supply chains:

(1) Introducing more subsidies to domestic pharmaceutical companies focusing on the production of new and innovative medicines, in order to gradually move from the market segment of common generics, characterized by over-capacity and low profit, and specialize in the high return production of quality drugs.

(2) Developing the supporting industries for pharmaceutical industry, particularly in the production of input materials, in order to narrow down the significant price gap between exported drugs from Vietnam and other countries.

(3) Keeping the domestic market open to foreign investors, particularly to producers, who have the intention to establish production factories in Vietnam. This not only could stimulate a reduction of the drug price in the domestic market but also paves the way for the establishment of productive linkages and spillovers between domestic and foreign firms.

## Figures and Tables

**Table 1:** Number of pharmaceutical establishments by type, 2010–2014.

Forms of Establishment	2010	2011	2012	2013	2014
Drug manufacturers achieving GMP *	101	109	119	123	131
Manufacturers of vaccines and biologicals achieving GMP	4	4	4	4	4
Manufacturers of finished herbal products achieving GMP	0	n/a	n/a	n/a	24 **
Units meeting GLP *	104	113	124	130	141
Importer-exporters achieving GSP *	0	n/a	n/a	n/a	174
Drug storage facilities meeting GSP	0	n/a	n/a	n/a	3
Drug-wholesalers achieving GDP *	0	n/a	n/a	n/a	1900
Retail drug outlet	43,629	39,172	39,124	42,262	n/a

* GMP: Good Manufacturing Practice standards; GLP: Good Laboratory Practice standards; GSP: Good Storage Practice standards; GDP: Good Distribution Practice standards (according to WHO or ISO 17025). ** 10 of these produce both herbal drugs and modern pharmaceuticals.

**Table 2:** List of biggest domestic pharmaceutical firms.

Company	Revenue Growth (2009–2013) (%)	Profit Growth (2009–2013) (%)	Total Turnover 2013 (mil. USD)
Hau Giang Pharmacy	19.2	13.1	168
Traphaco	22.5	35.3	80
Domesco	7.6	8.9	68
IMEXPHARM	6.3	−2	40
OPC	11	3.3	27
Cuu Long Pharmacy	4	−14.4	32
SPM	14.7	−27.2	21
Pharmedic	16.5	23.9	17
Phong Phu Pharmacy	19.3	n.a.	5

**Table 3:** Proportion of drugs sample that fail to meet quality standard, 2010–2013.

Year	Number of Samples Taken for Quality Assurance	Number of Samples Failed the Quality Tests	Percentage of Drugs Failed the Quality Test
2010	32,313	1008	3.12
2011	33,508	950	2.81
2012	32,949	1008	3.09
2013	39,482	948	2.54
2014	40,711	967	2.38
